# Assessment of Low Birth Weight and Associated Factors Among Neonates in Butajira General Hospital, South Ethiopia, Cross Sectional Study, 2019

**DOI:** 10.1155/2020/5841963

**Published:** 2020-07-25

**Authors:** Tigistu Toru, Walellign Anmut

**Affiliations:** ^1^Department of Nursing, Wolkite University, Ethiopia; ^2^Department of Midwifery, Wolaita Sodo University, Ethiopia

## Abstract

**Background:**

Low birth weight is defined as when a newborn weighs less than 2,500 grams within an hour of birth. Globally, it has been known that around 15.5% of newborns were below the normal level of weight at their birth and 95% of these infants lived in developing countries. The main objective of this study was to assess the prevalence and associated factors of low birth weight among newborns delivered at Butajira General Hospital, Southwest Ethiopia.

**Methods:**

An institutional-based cross-sectional study design was employed. All 196 paired study participants (newborn-mother) who were born on a one-month duration of the data collection period were included in the study. A pretested questionnaire was used to gather pertinent information about mother and newborn along with measuring newborn birth weight.

**Result:**

Majority of mothers 175 (92.1%) were aged between 20 and 34 years, and 186 (97.9%) were married. About 169 (88.9%) were protestant religion followers. This study showed that the magnitude of low birth weight among study participants was 12.5%, and factors such as maternal medical complication during pregnancy, maternal MUAC less than 23 cm, and birth interval less than 24 months were significantly associated with low birth.

**Conclusion:**

The study finding indicated that a significant number of newborns measured underweight which is below the normal level of weight at birth. The study identified factors such as maternal medical condition during pregnancy, maternal MUAC less than 23 cm, and birth interval less than 24 months. Based on study findings, we recommend health care officials, policymakers, key persons in the family, and volunteers to work on nutritional values particularly during pregnancy and before pregnancy. Spacing of birth is crucial to have healthy baby and healthy family even healthy society at large, so attention should be paid on family planning utilization.

## 1. Background

Low birth weight is defined by the World Health Organization (WHO) as weight at birth of less than 2,500 grams. Birth weight is an important indicator of health status of an infant and is a principal factor that determines the infant survival and physical and mental growth. It also indicates the past and present health status of mother [1, 2].

LBW is considered as a single most important predictor of infant mortality, especially of death within the first month of life [3]. It is also a significant determinant of infant and childhood morbidity particularly neurodevelopment impairment such as mental retardation and learning disabilities [4]. Low birth weight results in a shorter stature, and at the age of adulthood, those infants who are born with low birth weight are more likely to have brain development retardation, poor language development, and intellectual impairments [5].

Globally, neonatal mortality is 20 times more likely for LBW newborns when compared to normal weight newborns (2,500 gm-4,000 gm) and it increases sharply as birth weight (BW) decreases [6]. Newborns with low birth weight are more likely to have a health problem and slower development from immediately after birth to later life and suffer from an extremely high rate of mortality and morbidity from infectious disease and underweight, stunting, or wasting beginning in the neonatal period through childhood [7]. Low birth weight newborns usually need extra hospital care where there is a constant concern and uncertainty over future health outcomes [8].

Low birth weight a major public health issue in developing countries like Ethiopia. LBW leads to impaired growth of the infant resulting in a higher mortality rate and increased morbidity. LBW is also an important indicator of maternal and child health [9]. A low birth weight carries an increased risk of death on the newborns early in life or exposure to multiple health and development challenges later. The burden of immediate health problems on low birth weight newborns has been relatively poorly documented in many low-income countries with a national demographic survey [10]. Therefore, this study intended to fill the gap regarding low birth and associated factors among newborns in Butajira General Hospital, South Ethiopia.

### 1.1. Significance of the Study

This study will help to fill the information gap regarding prevalence and associated factors of LBW in Butajira General Hospital.

This study may increase the awareness of health professionals such as midwives, nurses, gynecologists, and others about the major risk factors for LBW and help them to give a proper and evidence-based health service (especially before and during pregnancy) in order to anticipate the problems associated with low birth weight.

Furthermore, this study will provide valuable information for researchers for further study.

## 2. Objectives of the Study

### 2.1. General Objective

To assess the prevalence and associated factor of low birth weight among newborns at Butajira General Hospital, South Ethiopia.

### 2.2. Specific Objectives

1. To determine the prevalence of low birth weight among newborns at Butajira General Hospital
2. To identify factors associated with LBW among newborns at Butajira General Hospital

## 3. Methods

### 3.1. Study Area and Study Period

This study was conducted in Butajira General Hospital found in Gurage Zone, South Ethiopia. Butajira is a town and separate woreda in south-central Ethiopia. Located at the base of the Zebidar mass in the Gurage Zone, Southern Nations, Nationalities and Peoples' Region. The Town has an elevation of 2,131 meters above sea level. It is surrounded by Meskane woreda.

Butajira General Hospital is situated 130 km south of the capital of Ethiopia, Addis Ababa, and 50 km to the west of Ziway town in the Rift Valley.

The study was conducted from May 1, 2019, to June 1, 2019.

### 3.2. Study Design

The institution-based cross-sectional study design was conducted from May 1, 2019, to June 1, 2019.

### 3.3. Population

#### 3.3.1. Source Population

All mother-newborn pairs in Butajira General Hospital during the data collection period were the source population.

#### 3.3.2. Study Population

The study population included selected mother-newborn pairs.

### 3.4. Eligibility Criteria

#### 3.4.1. Inclusion Criteria

All mothers who delivered live newborns during the study period were included in the study.

### 3.5. Sample Size Determination

The sample size was calculated by using a single population proportion formula by taking the prevalence of LBW which was 13.4% [20] and margin of error = 5%, confidence level = 95%, and standard normal distribution value = 1.96.

A total sample size was determined as follows: *n* = (*Z* *a*/2)^2^*p* (1 − *p*)/*d*^2^, where *n* is the desired sample size, *d* is the desired precision 5% = (0.05), *z* is the standard normal distribution value at confidence level 95% = 1.96, *p* is the prevalence rate of low birth weight = 13.4%, (*Zap*/2)^2^ = (1.96)^2^ = 3.8416, *P* = 13.4% = 0.134, and *d* = (0.05)^2^ = 0.0025.

So, *n* = 3.8416∗0.134∗0.866/0.0025 = 178.32.

By taking nonresponse rate, 10% of 178.32 = 17.832.

The total sample size was 178.32 + 17.832 = 196.15 ~ 196.

### 3.6. Sampling Technique

A simple random sampling technique was used to select study participants.

### 3.7. Variables of the Study

#### 3.7.1. Dependent Variable

The dependent variable is low birth weight.

#### 3.7.2. Independent Variables

- Sociodemographic characteristics: maternal age, family monthly income, educational level, maternal occupation, religion, marital status, ethnicity, and residence
- Maternal medical and obstetric characteristics: gravidity, parity, history of abortion, desirability of pregnancy, history of previous preterm birth, birth interval between current and previous last pregnancy, HIV status of the mother, ANC follow-up, number of ANC visits, and medical complication during pregnancy like malaria, PIH, and anemia
- Nutritional status of mother: average daily food consumption, additional diet during pregnancy, iron and folic acid supplementation during pregnancy, nutritional counseling during pregnancy, height of the mother, and MUAC of the mother
- General maternal behaviors: history of alcohol drinking, cigarette smoking, and chewing chat
- Newborn characteristics: sex of the newborn, gestational age at birth, and birth order

### 3.8. Operational Definition

Low birth weight is a weight of less than 2,500 g (up to and including 2,499 g) irrespective of the gestational age [36].

Gestational age is the duration of time measured from the first day of conception and expressed in completed weeks.

Preterm birth is the birth of newborn before 37 completed weeks of gestational age.

Gravidity is the number of pregnancy.

Parity is the number of births or the number of children either alive or died.

Birth order is the sequence at which birth occurs.

Maternal behavior is a habit of smoking cigarette, drinking alcohol, and chewing chat.

Birth interval is a time period between the current and previous last pregnancy.

### 3.9. Data Collection Technique

Data was collected by using a pretested, semistructured questionnaire with an interview type of data collection method, reviewing records from medical registration cards and actual measurement of some variables. Weight of newborn was measured immediately after delivery by using the neonate weight measurement scale. The questions were prepared in English and translated into Amharic language. The interview was in Amharic language, commonly used local language.

### 3.10. Data Quality Control

The quality of data was ensured during collection, coding, entry into the computer, and analysis. Orientation was given to data collectors and supervisors. Nurses who are currently working are involved in data collections. Each mother was asked, and the cards also were checked for consistency, provision of full information, and appropriate documentation. Neonate birth weight was measured using a birth weight measurement scale which is usually used in a hospital; its measuring accuracy was checked by supervisors before actual measurement takes place. The questionnaire was pretested on a 5% sample size before data collection using women postnatal room. The filled questionnaire was cross-checked by team members for completeness and closely supervised by supervisors daily.

### 3.11. Data Processing and Analysis

Data were entered by using epi-data version 3.1, and SPSS version 24 was used for analysis. Logistic regression was used to see the association between an outcome variable and independent predictors.

## 4. Result

All 196 mothers with their paired newborns responded which gives a response rate of 100%. Majority of mothers 175 (92.1%) were aged between 20 and 34 years, and 186 (97.9%) were married. About 169 (88.9%) were protestant religion followers. Nearly above half of 107 (56.3%) had a monthly income of 3,000 Ethiopian birr, 124 (65.3%) lived in a rural area, 86 (45.3%) were housewives, and 67 (35.3%) were educated grade 1-8 (primary school).

### 4.1. Medical, Obstetrics, Behavioral, and Nutritional Characteristics

Most of the mothers 184 (96.8%) responded that their pregnancy was planned, and 180 (94.7%) were tested for HIV status and declared as negative. About 136 (71.6%) were gravid 2 to 5, 141 (74.2%) were Para 2 to 5, 80 (40.5%) had birth interval less than 24 months, and about 29 (19.0%) and 10 (6.5%) had a history of abortion and preterm birth, respectively. About 184 (96.8%) mothers had ANC follow-up, and from these, only 49 (26.5%) mothers had ANC visit greater or equal to four visits, 54 (29.4%) mothers had no iron and folic acid supplementation, and 46 (25.0%) mothers had no nutritional counseling during current pregnancy. About 129 (67.9%) mothers did not consume additional diet than usual during current pregnancy, and about 120 (61%) and 100 (51%) mothers had a height greater than or equal to 155 cm and midupper arm circumference less than or equal to 23 cm, respectively. In this study, about 50 (25%) mothers had a history of medical conditions, and from these, 14 (28%) mothers had hypertension, 12 (24%) had Anemia, 15 (30%) had malaria, and 11 (22%) mothers had other medical conditions. All mothers 196 (100%) participated in this study had no habit of cigarette smoking, alcohol drinking, and chewing chat.

### 4.2. Newborn Characteristics

Of the total 196 newborns, 100 (51.6%) were female. Most of them 185 (94.4%) were born at a gestational age greater than or equal to 37 completed weeks. About 123 (64.6%) newborns were 2 to 5 birth order, and about 23 (12.5%) newborns were born with weight less than 2,500 grams.

### 4.3. Factors Associated with Low Birth Weight

According to this study finding, birth interval between current and previous last pregnancy, maternal medical conditions, and maternal midupper arm circumference were factors significantly associated with LBW at *p* < 0.0001 (Table 1).

## 5. Discussion

Birth weight is an important indicator of the health status of newborn, and it is a principal factor that determines infant physical, survival, and developmental stages. It also indicates the past and present health status of the mother [2]. The result of this study showed that 12.5% were measured as LBW at the time of birth. WHO and UNICEF estimated the prevalence of LBW about 15.5% worldwide, 15% in sub-Saharan Africa, and 11% in Kenya [12]. Another study done in India showed the prevalence of LBW was 11% [14]. The WHO country cooperation strategy 2008–2011 showed that the prevalence of low birth weight in Ethiopia was 14% [13]. According to the Ethiopian Demographic and Health Survey (EDHS 2011), 11% of newborns weighed less than 2,500 grams [23]. A survey conducted in Jimma hospital showed that out of 1,441 live births, 147 (10.2%) neonates weigh less than 2,500 gram [17]. A study conducted on birth weight in Gondar town documented the prevalence of LBW to be 13.4% [20]. Another study conducted to determine the prevalence of LBW in all hospitals in Addis Ababa reported at a rate of 12.6% [22]. We observed a slightly lesser proportion of LBW in the current study as compared to the above studies. The variation might be due to accessibility of health facility, number of health professionals and training for health professionals, prenatal care for pregnant mothers, supplementation of micronutrient (iron and folic acid) for pregnant women, family planning method for prevention of unwanted and unplanned pregnancy, ANC follow-up, and sociodemographic variations. Conversely, we found a higher proportion of LBW in our study as compared to the proportion of LBW estimated by Kenya Demographic Health Survey (KDHS 2009) (6%) [18], and the study done in Dessie hospital was 7.2% [22].

According to this study, mothers who gave birth for a birth interval less than 24 months between the last and current pregnancy were eleven times at an increased risk to give LBW when compared with having a birth interval greater than 24 months (AOR = 11.125, 95%CI = 2.008, 15.646). This study finding is consistent with a community-based study done in Karaka, India [25], and also with the finding in Zimbabwe [26]. Mothers who had medical conditions during current pregnancy were ten times at a higher risk of giving LBW newborns (AOR = 10.419, 95%CI = 1.012, 17.238) when compared with mothers who had no medical conditions during the current pregnancy. This finding was in line with the studies conducted in Jimma hospital [17], a study done in Tanzania [27], and a study done in India [31].

Another factor identified in this study was mother MUAC of a mother. Mothers whose midupper arm circumference measurement is 23 cm and above were at a lower risk of giving LBW newborns (AOR = 0.17, 95%CI = 0.01, 0.321) than mothers who had midupper arm circumference less than 23 cm. This finding was in consistent with the study done in Addis Ababa [24].

## 6. Limitation of the Study

Since this study used a cross-section study design which could not show a cause-effect relation between variables, measuring newborn weight may induce bias of accuracy. The study used a small sample size due to the availability issues of participants.

## 7. Conclusion

The study indicated that the magnitude of low birth weight among participants was high. Factors associated with LBW were maternal medical conditions, maternal midupper arm circumference, and birth interval between previous last pregnancy and the current one. Based on study findings, we recommend health sector officials, professionals, and government and nongovernment organizations to work on maternal nutritional status which directly affects newborn weight. In addition, keeping recommended birth spacing is required to advance both mother and infant.

## Figures and Tables

**Table 1:** Factors associated with low birth weight among newborns delivered at Butajira General Hospital, Southern Ethiopia, March 2019 (*n* = 196).

Variables	Categories	LBW		COR (95% CI)	AOR (95% CI)
		Yes (%)	No (%)		
History of abortion	Yes	6 (20.7)	23 (79.3)	3.333 (1.081, 10.276)	2.183 (0.397, 11.9961)
	No	9 (7.3)	115 (92.7)	1	1
Birth interval	<24 months	6 (37.5)	10 (62.5)	8.533 (2.527, 28.819)	**11.125 (2.008, 15.646)** ^∗^
	24 months & above	9 (6.6)	128 (93.4)	1	1
History of preterm birth	Yes	3 (30.0)	70 (70.0)	0.214 (0.049, 0.935)	6.89 (0.328, 141.332)
	No	12 (8.4)	131 (91.6)	1	1
Height of mother	<155 cm	4 (21.1)	15 (78.9)	2.773 (0.816, 9.428)	0.226 (0.006, 8.288)
	155 cm & above	15 (8.8)	156 (91.2)	1	1
MUAC of mother	<23 cm	3 (37.5)	5 (62.5)	1	1
	23 cm & above	16 (8.8)	166 (91.2)	6.225 (1.361, 28.477)	**0.17 (0.01, 0.321)** ^∗^
Medical conditions	Yes	4 (33.3)	8 (66.7)	5.433 (1.464, 20.168)	**10.419 (1.012, 17.238)** ^∗^
	No	15 (8.4)	163 (91.6)	1	1
Iron & folic acid	Yes	5 (3.8)	125 (96.2)	0.140 (0.047, 0.421)	0.176 (0.031, 1.001)
	No	12 (22.2)	42 (77.8)	1	1
GA at birth	<37 week	2 (40.0)	3 (60.0)	6.588 (1.028, 42.213)	2.326 (0.039, 137.759)
	37 week & above	17 (9.2)	168 (90.8)	1	1

^∗^Statistically significant at *p* < 0.05.

## Data Availability

The data used to support the findings of this study are available from the corresponding author upon request.

## Abbreviations

ANC: Antenatal care
AOR: Adjusted odds ratio
BW: Birth weight
CI: Confidence interval
COR: Crude odds ratio
EDHS: Ethiopian Demographic and Health Survey
ETB: Ethiopian birr
ELBW: Extremely low birth weight
GA: Gestational age
LBW: Low birth weight.

## References

[1] United Nation Children Fund (2012). *Low birth weight; country, regional and global*.

[2] Ramankutty P., Tikreeti R. A. S., Rasaam K. W., Al-thamery D. M., Yacoub A. A. H., Mahmood D. A. (1983). A Study on Birth Weight of Iraqi Children. *Journal of Tropical Pediatrics*.

[3] Viengsakhone L., Yoshida Y., Harun-Or-Rashid M., Sakamoto J. (2010). Factors affecting low birth weight at four central hospitals in vientiane, Lao PDR. *Nagoya journal of medical science*.

[4] Chiarotti F., Castignani A. M., Puopolo M., Menniti-Ippolito F., Minniti de Simeonibus E., di Paolo A. (2001). Effects of socio-environmental factors on neurocognitive performance in premature or low-birth weight preschoolers. *Annali dell'Istituto superiore di sanità*.

[5] Lawn J. E., Cousens S., Zupan J. (2005). 4 million neonatal deaths: when? Where? Why?. *Lancet*.

[6] Badshah S., Mason L., McKelvie K., Payne R., Lisboa P. J. G. (2008). Risk factors for low birthweight in the public-hospitals at Peshawar, NWFP-Pakistan. *BMC Public Health*.

[7] Kahsay Z., Tadese A., Nigusie B. (2014). Low Birth Weight & Associated Factors Among Newborns in Gondar Town, North West Ethiopia: Institutional Based Cross- Sectional Study. *Journal of Pharmaceutical Sciences*.

[8] Roudbari M., Yaghmaei M., Soheili M. (2007). Prevalence and Risk Factors of Low-Birth-Weight Infants in Zahedan, Islamic Republic of Iran. *Eastern Mediterranean Health Journal*.

[9] Shashikantha S. K., Sheethal M. P. (2016). Prevalence of low birth weight and its associated factors: a community based cross sectional study in a rural area of Rohtak, Haryana, India. *International Journal Of Community Medicine And Public Health,*.

[10] Sutan R., Mohtar M., Mahat A. N., Tamil A. M. (2014). Determinant of low birth weight infants: a matched case control study. *Open Journal of Preventive Medicine*.

[11] Gessessew B., Balem D., Mussie A. (2014). Socio-demographic and maternal determinants of low birth weight at Mekelle Hospital Northern Ethiopia, a cross-sectional study. *American Journal of Advanced Drug Delivery*.

[12] United Nations Children Fund (2004). *LBW, country; regional and global*.

[13] WHO nutrition and food safety WHO country cooperation strategy 2008-2011, Ethiopia.

[14] Tabrizi F. M., Saraswathi G. (2012). Maternal anthropometric measurements and other factors: relation with birth weight of neonates. *Nutrition Research and Practice*.

[15] US Department of Health & Human Service (2011). *Health Resource and Service Administration, Maternal and Child Health Bureau, Child Health*.

[16] Tema T. (2006). Prevalence and determinants of low birth weight in Jimma zone, Southwest Ethiopia. *East African Medical Journal*.

[17] Gebremariam A. (2006). Factors predisposing to low birth weight in Jimma Hospital South Western Ethiopia. *East African Medical Journal*.

[18] Kenya National Bureau of Statistics (KNBS) (2010). *Kenya Demographic and Health Survey 2008-09*.

[19] WHO (2006). *The Indicator of Low Birth Weight; An update Epidemiology, Rec1984: 59*.

[20] Young P. N. (2007). Birth weight in 60 hospital delivered infant in Addis Ababa and Gondar. *Ethiopia Medical Journal.*.

[21] Zein A. Z. (1991). The distribution of low birth weight in the Gondar administrative region, north-west Ethiopia. *Ethiopian Journal of Health Development*.

[22] Green-Abate C. (1986). Changes in birthweight distribution from 1973 to 1982 in Addis Ababa. *Bull World Health Organ*.

[23] Ethiopian Demographic and Health Survey.

[24] Yihdego M. (2014). *Assessment of maternal risk factors associated with full-term low birth weight neonates in public health facilities of Addis Ababa, Ethiopia: a case-control study*.

[25] Metgud C. S., Naik V. A., Mallapur M. D. (2012). Factors Affecting Birth Weight of a Newborn – A Community Based Study in Rural Karnataka, India. *Plos One*.

[26] Mbuya M. N. N., Chidem M., Chasekwa B., Mishra V. (2010). *Biological, Social and Environmental Determinants of Low Birth Weight and Stunting among Infants and Young Children in Zimbabwe*.

[27] Siza J. E. (2008). Risk factors associated with low birth weight of neonates among pregnant women attending a referral hospital in northern Tanzania. *Tanzania Journal of Health Research*.

[28] Lowa *Factors associated with infant low birth weight among Medicaid reimbursed births in Iowa, 2010*.

[29] Dasgupta A., Basu R. (2011). Determinants of low birth weight in a Block of Hooghly, West Bengal: A multivariate analysis. *International Journal of Biological & Medical Research*.

[30] Agarwal G. (2012). Maternal Risk Factors Associated with Low Birth Weight Neonates in a Tertiary Care Hospital, Northern India. *Journal of Community Medicine & Health Education*.

[31] Mumbare S. S., Maindarkar G., Darade R., Yenge S., Tolani M. K., Patole K. (2012). Maternal risk factors associated with term low birth weight neonates: A matched-pair case control study. *Indian Pediatrics*.

[32] Kumar S. G., Kumar H. N. H., Jayaram S., Kotian M. S. (2010). Determinants of low birth weight: A case control study in a district hospital in Karnataka. *The Indian Journal of Pediatrics*.

[33] Rizvi S. A., Hatcher J., Jehan I., Qureshi R. (2007). Maternal risk factors associated with low birth weight in Karachi: a case-control study. *Eastern Mediterranean Health Journal*.

[34] Strauss R. S., Dietz W. H. (1999). Low Maternal Weight Gain in the Second or Third Trimester Increases the Risk for Intrauterine Growth Retardation. *The Journal of Nutrition*.

[35] Lawoyin T. O. (1997). The relationship between maternal weight gain in pregnancy, hemoglobin level, stature, antenatal attendance and low birth weight. *Southeast Asian J Trop Med Public Health*.

[36] United Nations Children’s Fund (2004). *Low birth weight: country, regional and global estimates*.

